# Analysis of the reasons for consultation in psychiatric emergency triage

**DOI:** 10.1192/j.eurpsy.2024.1266

**Published:** 2024-08-27

**Authors:** S. Cruz, S. R. Ferre, F. V. Español

**Affiliations:** ^1^Hospital Universitario de Jaen, Jaén; ^2^ Hospital Universitario de Jaen; ^3^Hospital Unviersitario de Jaén, Jaen, Spain

## Abstract

**Introduction:**

The chain of care in psychiatric emergencies should be reviewed to improve assistance.

**Objectives:**

Our objective was to determine the reality behind the reasons for consultation assigned in triage as “Psychiatry Assessment” and “Psychiatric Patient”, examining diagnoses to the discharge of said patients

**Methods:**

To this end, reasons for triage consultation and patient diagnoses are retrospectively collected who were evaluated by the main author in the emergency room of Hospital de Jaén between June 23, 2019 and May 31, 2020. They were selected following these criteria; inclusion: patients with psychiatry consultation, evaluated by the first signatory of the text and with reasons for consultation in triage: “Psychiatric patient” or “Assessment by Psychiatry”. As exclusion criteria: high due to escape. Among the 224 patients evaluated, we found 35 who met criteria

**Results:**

Of the total reasons of consultation collected at beginning, 16.6% corresponds to “Assessment by Psychiatry” (13.9%) and “Patient psychiatric” (2.7%), this being group the second reason for most frequent consultation after of “Anxiety” with 33%. Relating these reasons for consultation with the discharge diagnoses made in these patients, we found that the percentage of patients in each diagnosis would be: Regarding the action plan followed after the evaluation and diagnosis of these patients, it is reported that 45% of them required admission, 37% were referred to Mental Health Unit, 9% to family doctor and 6% to the Drug Addiction Center. - 11.4% of pharmacological intakes; 8.6% of psychotic episodes, symptoms anxiety, treatment renewal and mood disorders personality; respectively; 5.7% of autolytic attempts, autolytic ideation, schizoaffective disorder, bipolar disorder, heteroaggressiveness and depression; respectively; 2.9% of adverse effects to drugs among others diagnostics

**Conclusions:**

It is appreciated that the reasons for consultation triated as “Psychiatric patient” or “Psychiatry assessment” does not provide real information about the clinical characteristics of the patient to be evaluated in the emergency room, having a wide range of diagnoses encompassed in these terms. This fact does not allow discern the fundamental reason why the patient goes to the emergency room, nor receive assistance adequate to the problem it presents, nor a correct regulation of waiting and logistical planning. We believe it is advisable to review the use of these terms in the practice of the psychiatric emergencies training all professionals involved in the triage chain and we value the need to count on all emergency services with a standardized triage method for the psychiatric emergencies.

**Disclosure of Interest:**

None Declared

